# Nano-Graphene Layer from Facile, Scalable and Eco-Friendly Liquid Phase Exfoliation Strategy as Effective Barrier Layer for High-Performance and Durable Direct Liquid Alcohol Fuel Cells

**DOI:** 10.3390/molecules27093044

**Published:** 2022-05-09

**Authors:** Prabhuraj Balakrishnan, Fereshteh Dehghani Sanij, Zhixin Chang, P. K. Leung, Huaneng Su, Lei Xing, Qian Xu

**Affiliations:** 1 Institute for Energy Research, Jiangsu University, Zhenjiang 212013, China; prabhuraj@ujs.edu.cn (P.B.); dehghan82@gmail.com (F.D.S.); zhixinchang1996@163.com (Z.C.); suhuaneng@ujs.edu.cn (H.S.); 2 MOE Key Laboratory of Low-Grade Energy Utilization Technologies and Systems, Chongqing University, Chongqing 400030, China; leungpuiki@hotmail.com; 3 Department of Chemical Engineering, Loughborough University, Loughborough LE11 3TU, UK; xinglei1314@gmail.com

**Keywords:** liquid alcohol fuel cells (LAFCs), membrane electrode assemblies (MEAs), graphene, liquid phase exfoliation, membrane selectivity

## Abstract

Graphene, in spite of exceptional physio-chemical properties, still faces great limitations in its use and industrial scale-up as highly selective membranes (enhanced ratio of proton conductivity to fuel cross-over) in liquid alcohol fuel cells (LAFCs), due to complexity and high cost of prevailing production methods. To resolve these issues, a facile, low-cost and eco-friendly approach of liquid phase exfoliation (bath sonication) of graphite to obtain graphene and spray depositing the prepared graphene flakes, above anode catalyst layer (near the membrane in the membrane electrode assembly (MEA)) as barrier layer at different weight percentages relative to the base membrane Nafion 115 was utilized in this work. The 5 wt.% nano-graphene layer raises 1 M methanol/oxygen fuel cell power density by 38% to 91 mW·cm^−2^, compared to standard membrane electrode assembly (MEA) performance of 63 mW·cm^−2^, owing to less methanol crossover with mild decrease in proton conductivity, showing negligible voltage decays over 20 h of operation at 50 mA·cm^−2^. Overall, this work opens three prominent favorable prospects: exploring the usage of nano-materials prepared by liquid phase exfoliation approach, their effective usage in ion-transport membrane region of MEA and enhancing fuel cell power performance.

## 1. Introduction

A massive increase in population, day-to-day technological improvements and their related projects, in this era have prompted exponential needs in our energy supply [1,2,3]. In addition, multiple circumstances: increased greenhouse gas emission, energy production costs, trade policies and shortage of conventional fuels (coal, oil and natural gas) have moved the researchers to look for novel energy devices [1,2]. The intense advantages of fuel cells are diverse: reduced detrimental effect on the atmosphere, few moving components, zero noise, and simple operational mechanisms, make them the most favorable in a wide pool of energy conversion and storage devices (batteries, capacitors and supercapacitors) to achieve the above mentioned goals [1,2,3]. Among different types, direct-liquid alcohol fuel cells (LAFCs) operating on methanol, ethanol and propanol, are favored due to their flexibility in the usage of liquid fuel (rather than hydrogen which experiences difficulties in transportation and storage), ambient temperature operation, high energy density of methanol and increased usage in portable electronic systems [3,4,5].

In spite of LAFC’s advantages, its commercialization is greatly hindered by excess fuel crossover (or permeability) issues degrading power performance at both short and long time-scale operations [5,6]. Fuel crossover is the permeation of methanol from anode to cathode of the fuel cell through the membrane, propelled by the combined effect of diffusion and electro-osmotic drag coefficient [6,7,8]. This permeated fuel at the cathode in the presence of catalyst leads to mixed-potential and short-circuit overall, degrading its efficiency and durability [6,7]. Therefore, reducing the methanol crossover with an aim to improve the power performance is the motive of this study.

To reduce the impact of this crossover issue so as to improve the power performance of the fuel cell, a barrier layer is applied between the anode electrode and membrane region in their membrane electrode assembly (MEA) [8,9]. Previously materials like: mordenite, polybenzimidazole, clay, zeolite, chitosan biopolymer, palladium, platinum nanowire, polyaniline, sulphonated polystyrene employed by spin-coating, sputtering and electrochemical deposition methods were reported so far [10,11,12]. All of them lowered fuel permeability values and resulted in higher power output than the standard ones [9,10]. However, the proton conductivity route was also affected in these works, so a balance between fuel crossover and conductivity were made to achieve power performance improvement [10,11]. Therefore, there is a still need for membranes with high selectivity (ratio of proton conductivity to fuel crossover ratio) to maximize performance improvements.

From a materials perspective, graphene, the first in the 2D materials family gets enhanced importance due to its exceptional physio-chemical properties: high thermal conductivity (5000 W·m^−1^·K^−1^), mechanical stability (Young’s modulus of 1 TPa), electron mobility (250,000 cm^2^·V^−1^·s^−1^), surface area (2630 m^2^·g^−1^) and optical transparency (97.7%) [13,14]. Especially its carbon–carbon bond length of 0.142 nm—rendering ability to block all atoms of size greater than helium is of significant interest for use as methanol barrier layer in fuel cells [15,16]. This was experimentally shown by Nair et al. [17] and Su et al. [18] where graphene membrane (graphene oxide and reduced graphene oxide prepared by Hummer’s method followed by chemical reduction) has impeded: propanol, decane, hexane ethanol, acetone, formic acid and gases: argon, hydrogen and nitrogen.

Furthermore, Hu et al. [19] have shown that single-layer graphene membranes (prepared by mechanical exfoliation) are prone to hydrogen ionic (protons) transport. These features of resisting liquid permeation, at the same time allowing ionic transport, show tremendous potential for use in liquid alcohol operated fuel cell membrane area [6,7,8].

This concept was utilized in an actual fuel cell system by Holmes et al. [20] and Yan et al. [21] as a barricade layer, respectively. Holmes et al. [20] used chemical vapor deposited (CVD) graphene transferred onto fuel cell anode and obtained 45% improvement in performance in active methanol fuel cell conditions. They reported different samples with various improvements, due to the difference in coverage of graphene over the electrode, caused during the transfer process. Meanwhile, Yan et al. [21] obtained 120% performance improvement at high concentration methanol passive fuel cell systems. However, these works reported a major drawback of surface coverage of graphene over the electrode, which is caused due to practical difficulties in graphene transfer from CVD furnace to the electrode and preparation methodology.

Xu et al. [22] used graphene-Nafion dispersion on the anode side and obtained 82% improvement at 8 M methanol and oxygen (60 °C cell temperature) conditions, where the graphene used were purchased from XFNANO Inc.

Considering the drawbacks of all the previous reports and in an effort to widen the horizons of this research, in this work we have used graphene from liquid phase exfoliation–bath sonication of graphite and utilized them as a barrier layer—(above the anode catalyst layer) showing an effective membrane with high selectivity (proton conductivity to fuel crossover ratio) for performance improvements in various LFACs—methanol, ethanol and propanol, respectively.

Liquid phase exfoliation involves dispersion of the precursor in a solvent (*N*-methyl pyrrolidone (NMP), acetone, dimethyl sulphoxide (DMSO), 1,2-dichloro benzene (DCB), acetophenone, water, benzonitrile, 1,4-dioxane) and subjecting to exfoliation by bath sonication [23,24]. This method offers lesser complexity, when compared to CVD (requirement of high temperature/high pressure furnace operation), electrochemical exfoliation and mechanical exfoliation (low percentage yield of few layers of graphene and heavy manual work) [23,24,25,26].

From the scalability point of view, 2D materials dispersed in solvents in high volumes, can be transported and utilized without losing its properties for prolonged timelines [23,24]. In addition, it is known that no gaseous side products during liquid exfoliation process are released, unlike chemical reduction, where oxides of nitrogen and sulphur are released at chemical exfoliation of 3D graphite to form graphite oxide and further reduction steps [25,26].

Furthermore, the graphene is deposited onto an anode fuel cell electrode (above the catalyst layer), using spray deposition, which is considered easier compared to the transfer process from furnace to electrode where CVD is used and the mechanical exfoliation method was used [27,28,29,30]. Taking into account various viewpoints—scalability, preparation simplicity and environmental friendliness of liquid exfoliation and further application by simple spray coating, this offers multiple advantages and is henceforth utilized in this work.

These 2D materials from liquid phase exfoliation methodology were reported to have been utilized in several opto-electronic applications (solar cells and thin film transistors), nano-sensors and nano-composites [23,24]. To the best of our knowledge, none have been used as a barrier layer in the MEA of fuel cells and is reported in this work. Among different solvent mediums for exfoliation, acetone is selected, due to its low boiling point and ease during fuel cell spray coating process features [28,29].

In the fuel cell membrane region, it is known that methanol permeability (MP) and proton conductivity are inter-related [6,7,8]. To evaluate this comprehensively, different weight (wt.%) percentages of graphene are employed above the anode catalyst layer as a barrier layer, due to its proximity to the membrane (in relation to weight of the base Nafion membrane). Coating onto the electrode is preferred, since approaches found in the literature: mounting barrier layer material directly onto membrane reported more complications: membrane swelling and membrane electrode assembly (MEA) damage [20,21,22].

The results are evaluated by materials characterization (atomic force microscopy (AFM), scanning electron microscopy (SEM), energy dispersive spectroscopy (EDS) with elemental mapping and Raman spectroscopy), electrode/membrane characterization—fuel permeability (MP—methanol permeability in case of methanol fuel usage) (electrochemical and gravimetric modes), water uptake, ion-exchange capacity, proton conductivity, fuel oxidation propensity (FOP)—oxidation current and peak oxidation potential, electrode reaction resistance (ERR), wettability and fuel cell polarization curves (open circuit voltage and peak power density values) at different fuel concentrations and temperatures.

Overall, this work of utilizing a liquid phase exfoliation approach of graphene synthesis, usage as a barrier layer on the anode side of MEA using simple spray deposition and with significant power performance increments obtained, will lead to advantages in multiple fronts: improving the commercial standpoint of LAFCs (efficiency and durability improvements as reported), influx of various liquid exfoliated 2D materials as high selective membranes into the fuel cell field and their further mechanism exploration studies.

## 2. Results and Discussion

### 2.1. Nano-Graphene Flake Characterization

**Figure 1:**
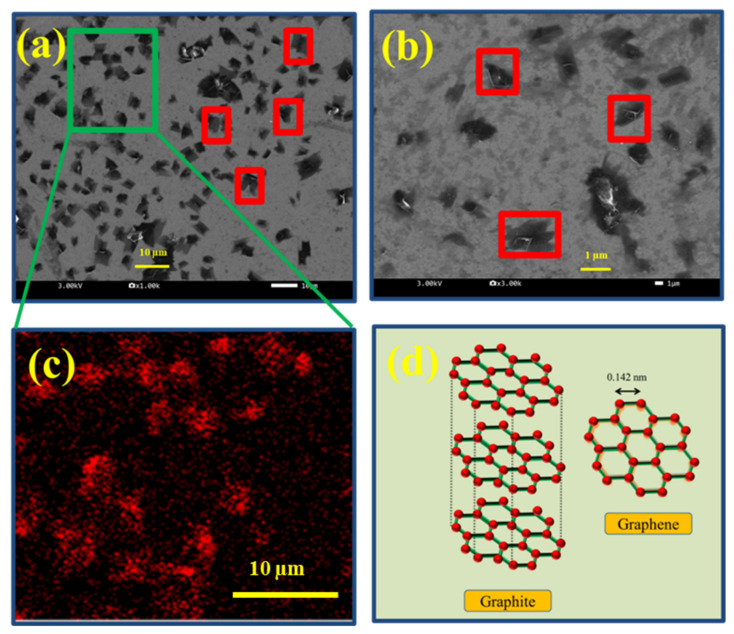
SEM images of graphene flakes drop casted on silicon wafer at (**a**) 10 µm magnification scale, (**b**) 1 µm magnification scale, (**c**) EDS mapping showing carbon and (**d**) illustration of graphite and graphene structures.

Figure 1a (10 µm magnification scale) and Figure 1b (1 µm magnification scale) shows the uniformly sized graphene flakes with lateral size: ~2–4 µm (few particles marked in red boxes), confirming the effectivity of the liquid exfoliation and centrifuge steps, as described in the experimental section. Furthermore, the electron dispersive spectroscopy (EDS)—mapping at Figure 1c, (indicative of green region in Figure 1a), further confirms the presence of carbon. Elemental analysis at different spots of this sample showed that the prepared graphene exhibited carbon to oxygen (C/O) ratio of 4.5 as given in Table S1 (Supplementary Materials), compared to a C/O ratio of 19.8 for graphite, which are in agreement with literature values [13,14,15].

The above results confirm the exfoliation of multiple carbon layers (40 µm thick) containing graphite onto a few layers of graphene with ~2–4 µm size, as illustrated schematically in the Figure 1d. The oxygen presence comes from the acetone ((CH_3_)_2_CO) medium exfoliation, as analyzed thoroughly by previous studies [14,15]. These oxygen groups are essential in obtaining spraying favorable dispersions, where they miscible with polar solvents [15,16]. Further importance of this C/O ratio (after spraying dispersions onto anode fuel cell electrode), altering hydrophilic/hydrophobic nature of electrodes and their role in affecting fuel cell performance are discussed in detail in the coming sections.

Figure S1 (Supplementary Materials) shows the Raman patterns of graphite and graphene on silicon wafer. It can be inferred that increased intensity of D-band (or disorder band) for graphene at 1300 cm^−1^, compared to graphite, indicates evaluation of defects and disorders in a sample arising from the exfoliation process [31,32]. The characteristic peak disappearance of the 2D-band (at 2700 cm^−1^) confirms that the material is of few layers [31]. These observations from D-band, G-band and 2D-band confirm the reduction in 325 mesh flake thick graphite (before exfoliation) to a few layers of graphene after the process, as previously illustrated in Figure 1d.

**Figure 2:**
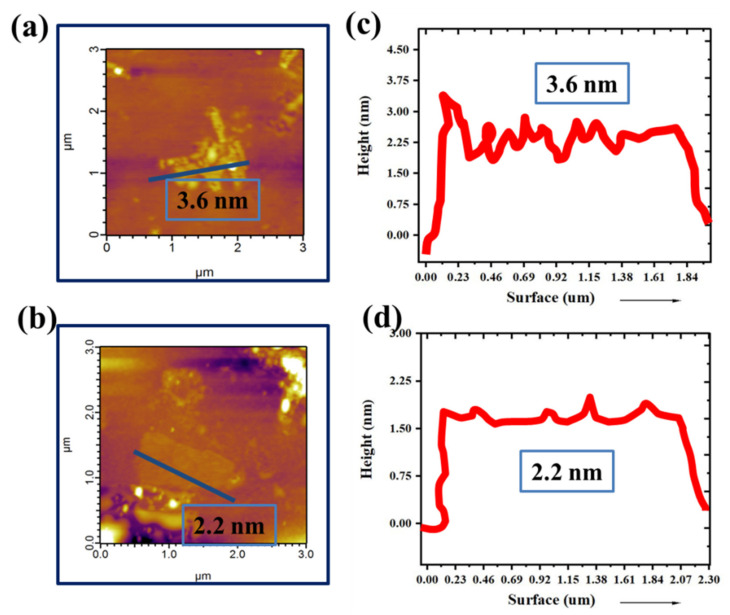
(**a**,**b**) Atomic force microscope (AFM) image and (**c**,**d**) thickness profile of graphene (drop casted on silicon wafers).

More precise information obtained from atomic force microscopy (AFM) and their corresponding surface profiles (marked in grey line) taken for different samples, shows that flake thickness is of 2–5 nm (Figure 2a–d) [33,34,35]. These characterizations agree well with the results in the followed graphene preparation procedure (Coleman et al. [36]).

### 2.2. Different Graphene wt.% Barrier Layer Characteristics—Electrode and Membrane Characterization Results

**Figure 3:**
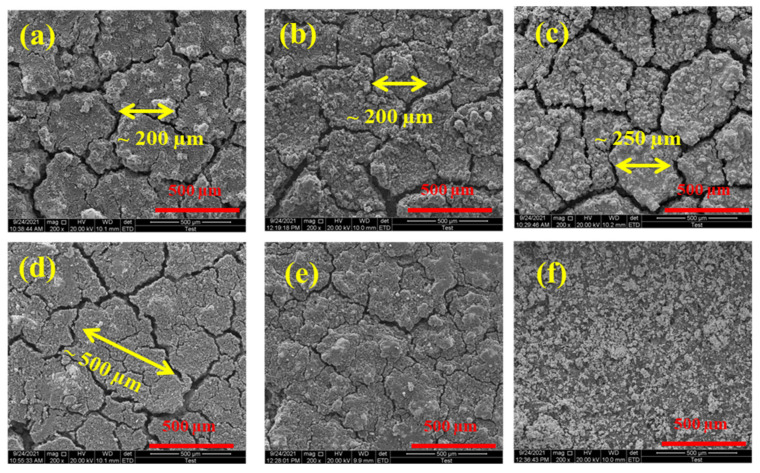
Top-view SEM images of electrodes—standard ((**a**) 0 wt.% graphene) and containing graphene ((**b**) 2.5 wt.% graphene, (**c**) 5 wt.% graphene, (**d**) 7.5 wt.% graphene, (**e**) 10 wt.% graphene and (**f**) 20 wt.% graphene) showing varied crack separation lengths.

**Figure 4:**
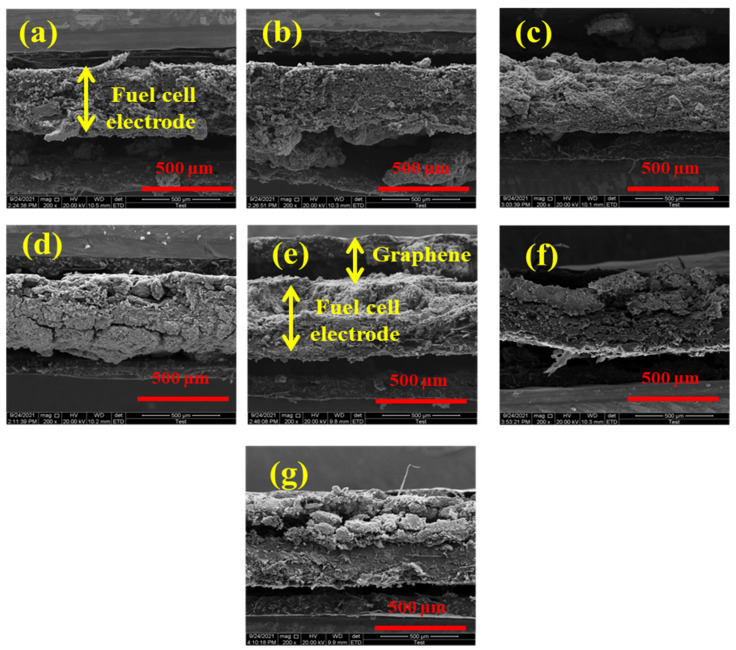
Cross-sectional SEM view of electrodes—standard ((**a**) 0 wt.% graphene) and containing graphene ((**b**) 2.5 wt.% graphene, (**c**) 5 wt.% graphene, (**d**) 7.5 wt.% graphene, (**e**) 10 wt.% graphene, (**f**) 20 wt.% graphene) and (**g**) 25 wt.% graphene showing varied crack separation lengths.

To precisely estimate the effect of graphene, this spray was deposited at: 2.5 wt.%, 5 wt.%, 7.5 wt.%, 10 wt.%, 20 wt.% and 25 wt.% values (0 wt.% graphene is noted as standard) on the anode electrode (as barrier above the catalyst layer as mentioned in the materials and methods section) and evaluated. Figure 3 and Figure 4 show the scanning electron microscope (SEM) images depicting changes in electrode structure as a result of adding a graphene layer. The uniform distribution of tiny graphene flakes over the catalyst layer of the fuel cell electrode is evident in the top-view SEM images in Figure 3a–f, confirming the reliability of spray coating.

It can be seen that the average crack separation length changed for the 5 wt.% graphene electrode (Figure 3c), compared to standard electrode, which is around 200 µm (as indicated in Figure 3a). On further increasing the graphene wt.%, the crack size enlarged (around 500 µm for 7.5 wt.% graphene layer as in Figure 3d), then nearly disappeared (for 10 wt.% graphene layer as in Figure 3e) and show agglomerated surface (for 12 wt.% graphene layer as in Figure 3f).

Changes in crack separation length are crucial, as previous studies by Hiramitsu et al. [37], Mohseninia et al. [38] and Li et al. [39] have attained improvements in hydrogen/methanol fuel cell performance by altering the microstructure of the electrode as a result of observing electrode crack size/porosity changes affecting water transport characteristics.

Since water transport changes also affect the methanol, which has direct influence on power performance [4,5,6], it can be inferred that changes in power performance are expected, (in addition to increased water uptake and ion-exchange capacity values) which are discussed in detail in the coming sections.

Figure 4 shows that the addition of graphene increased the thickness of the electrode (cross-sectional view) in proportion to the weight percentage of addition. From Figure 4a–c, graphene layer is not clearly visible due to minor increase in thickness, due to low percentage addition of graphene, whereas from Figure 4d–g, this becomes more clear than previous as indicated in Figure 4e.

Electrode porosity (Complete, hydrophobic—estimation of water repelling pores and hydrophilic—estimation of water affinity pores) by weight loss method offers enhanced simplicity compared to gas-liquid porosimetry methods, and pertains to methanol/water transport in their structure [38,39]. Similarly, contact angle measurements gives approximation of wettability of electrode characteristics towards water and plays a major role in relating cell performance and water transport through its pores [37,39]. 

Hence, electrode porosity and contact angle values of different electrodes are determined as explained in the experimental procedure and given in Table 1.

The hydrophobic porosity of electrodes increased with the graphene content, (60% to 73%), higher than that of standard electrode (60%). This is due to high carbon to oxygen (C/O) of 4.5 of the prepared graphene from electron dispersive spectroscopy (EDS) results, compared to platinum (and Nafion ionomer)-containing standard, which are well known for their water-affinity nature. The similar trend is observed in the contact angle measurements due to the above reasons. Studies by Bera et al. [40] and others have reported contact angle values of 180° for water on single-layer graphene surfaces. This high value is not observed in this case because of low percentage additions of graphene, with the maximum being 25 wt.% graphene showing 163° contact angle. Furthermore, the increase is gradual, in accordance with the increase of graphene content.

These changes in hydrophobicity and contact angle values of the electrodes, along with the crack separation (as observed earlier) are expected to play a role in the water/methanol repelling characteristics of the electrode (as previous studied well in literature) and it will be interesting to observe the power performance.

Water uptake (WU) and ion-exchange capacities characteristics of a MEA gives an understanding of proton conductivity since protons undergoes Grotthus mechanism movement through functional groups of the membranes [5,6,7]. Hence, these tests are carried out as explained in the experimental procedure and percentage difference (in relation to the standard MEA tabulated in Table S2, Supplementary Materials). 

From Table S2, it can be seen that WU decreased with increasing graphene wt.% content. This is in accordance with hydrophobic porosity and contact angle measurements, which implies that graphene with high C/O ratios repelled water. Whereas, ion-exchange capacity values increased with graphene wt.% content, indicating that ascending oxygen functional groups with the graphene wt.% increase has led to this value. 

Membrane characterizations results (methanol permeability (MP), electrochemical mode) and proton conductivity were carried out, as explained in the experimental section and tabulated in Table 2. Since better fuel cell power was observed for 1 M methanol and 60 °C cell temperature conditions, characterizations are carried out at these conditions. Furthermore, membrane selectivity factor has direct relation to the fuel cell performance; this is calculated and given in Table 2 as well.

It can be seen that MP, electrochemical mode values decrease with the increase in graphene weight percentage, showing that this graphene acted as a barrier layer, preventing fuel permeability thereby preventing undesirable fuel oxidation on the cathode. Meanwhile the proton conductivity has shown a declining trend, indicating that this layer though reduced MP has caused hindrance to proton conductivity. Hence, it can be inferred that the water-repelling nature of the graphene (as confirmed previously from hydrophobic porosity, water uptake and contact angle measurements) has resulted in reduced methanol permeation. 

Calculating membrane selectivity for all samples, the 5 wt.% graphene exhibited a high value of 0.77 mS·cm·mA^−1^, indicating this layer prevented fuel permeation at the same time minimally affecting proton transfer route.

### 2.3. Different Graphene wt.% Barrier Layer Characteristics—Fuel Cell Results

To test their effect in fuel cells, standard and graphene-containing membrane electrode assemblies (MEAs) are compared at 60 °C cell temperature, 1 M methanol (10 mL·min^−1^) and oxygen (250 mL·min^−1^) operating parameters, as best standard power performance is obtained in these conditions. 

On adding the graphene at 2.5 wt.% on the anode side, the peak power density improved from 63 mW·cm^−2^ for the standard to 73 mW·cm^−2^ (Figure S2a,b, Supplementary Materials).

**Figure 5:**
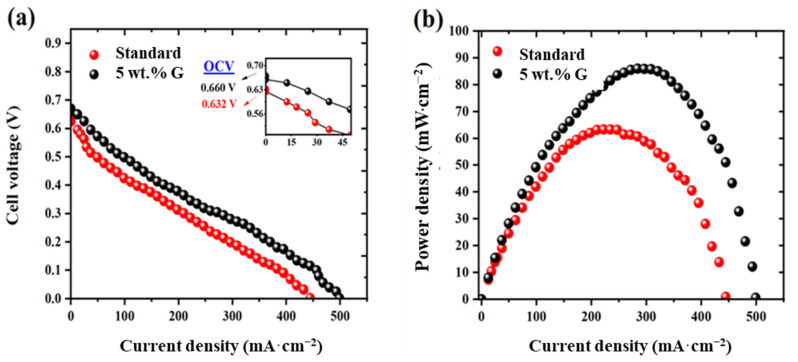
(**a**) Cell voltage and (**b**) power density profile obtained at 60 °C in 1 M methanol/oxygen conditions for standard and 5 wt.% graphene MEAs.

On increasing the graphene content at 5 wt.% (noted as 5 wt.% G in the Figure 5), the peak power density of 91 mW·cm^−2^ is attained (Figure 5a,b). The voltage profile (inset of Figure 5a) shows that the open circuit voltage (OCV) for graphene is 0.660 V, whereas for the standard is 0.630 V, indicating less methanol permeation. It is known that polarization curve consists of: activation (low current density), ohmic (middle region) and mass-transport (high current density) regions corresponding to interplay of methanol crossover, membrane conductivity and oxygen transfer phenomenon [2,3,9]. The increase in OCV confirms this effect. 

**Figure 6:**
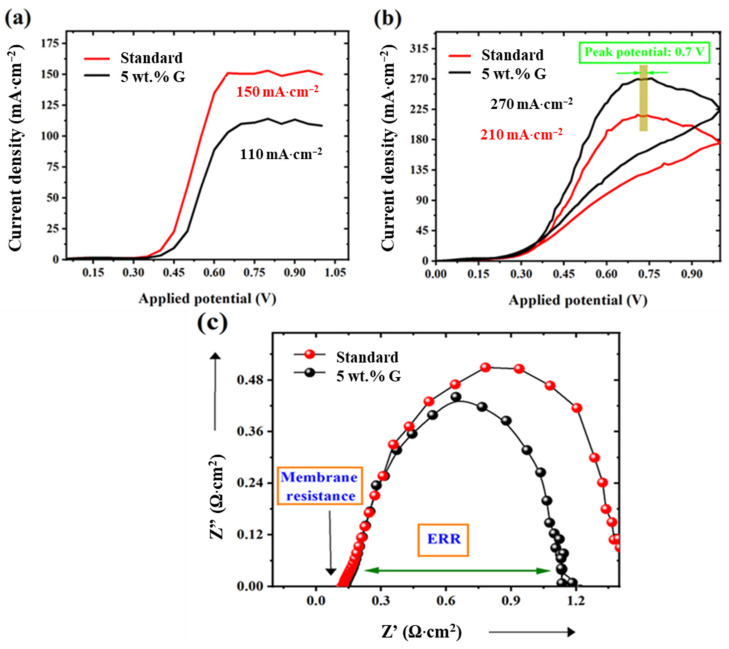
(**a**) Methanol crossover (by electrochemical mode), (**b**) methanol oxidation ability and (**c**) impedance spectroscopy results at 60 °C in 1 M methanol conditions for standard and graphene 5 wt.% electrodes.

Since high value of membrane selectivity of 0.77 mS·cm·mA^−1^ is obtained for this 5 wt.% graphene electrode as in previous discussions, this MEA is studied in detail. On characterization for membranes, the methanol permeation (MP, electrochemical, mode) has reduced to 110 mA·cm^−2^, compared to 150 mA·cm^−2^ for the standard (Figure 6a). 

This methanol blocking has led to better methanol utilization on the anode side, showing enhanced fuel oxidation propensity (FOP)—methanol oxidation current of 270 mA·cm^−2^ (graphene) than 210 mA·cm^−2^ for the standard (Figure 6b). Also, the similar peak potential at 0.7 V for both standard and 5 wt.% graphene, indicates negligible catalytic activity of graphene towards methanol oxidation.

Impedance spectroscopy plot shows that this 5 wt.% graphene barricade layer has reduced proton conductivity from 100 to 85·mS·cm^−1^—calculated from x-axis intercept (Figure 6c). However, this has not affected the overall fuel cell performance. This is due to decreased permeation and increased methanol oxidation effects being more dominant than the proton resistance effect. Furthermore, the ERR values for 5 wt.% graphene (1.1 Ω·cm^2^) are lower than the standard (1.35 Ω·cm^2^), (Figure 6c) reflecting fuel cell results, caused by the above factors.

MP results by gravimetric modes as shown in Figure S3a (Supplementary Materials) indicated less loss in weight for the 5 wt.% graphene electrode than the standard, thereby confirming previous electrochemical results. Figure S3b shows the electrode wettability of standard and 5 wt.% graphene, showing the hydrophobic nature of graphene compared to platinum surface.

On further increasing the graphene content above 5 wt.%, the performance drastically dropped from 63 mW·cm^−2^ (standard) to 45 mW·cm^−2^ for 10 wt.% graphene, then to 29 mW·cm^−2^ for 20 wt.% graphene. This is due to the dominant impact of proton resistance on cell performance (Table 2), though decreased methanol permeation effect is experienced, as listed in the Table S3, Supplementary Materials. 

**Figure 7:**
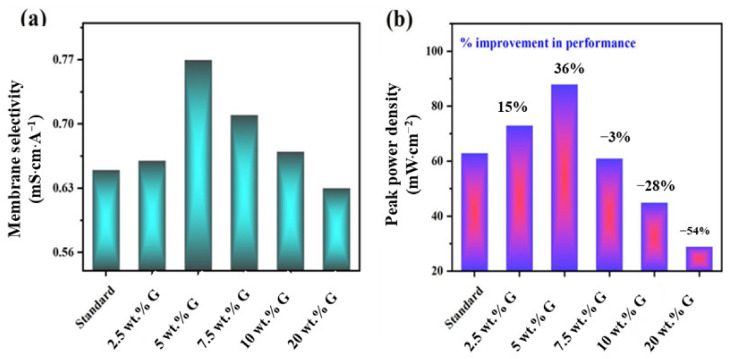
Plot of impact of graphene wt.% on (**a**) membrane selectivity and (**b**) peak power density at 1 M methanol and 60 °C cell conditions.

Therefore, taking into account all the materials, electrode and membrane characterization results as discussed before, it can be inferred that all these factors have aided in increased selectivity, thereby enhancing performance facilitated by 5 wt.% graphene layer as depicted in Figure 7a,b, showing influence of selectivity on performance for different samples.

### 2.4. Effect of Temperature on Fuel Cell Performance

This performance improvement was found to increase linearly with temperature showing that the 5 wt.% graphene barricade layer impeded methanol permeation, to a greater extent with slight resistance to protons at different temperatures. Figure S4 (Supplementary Materials) gives the peak power density at different temperatures.

### 2.5. High Concentration Methanol Fuel Cell Testing

**Figure 8:**
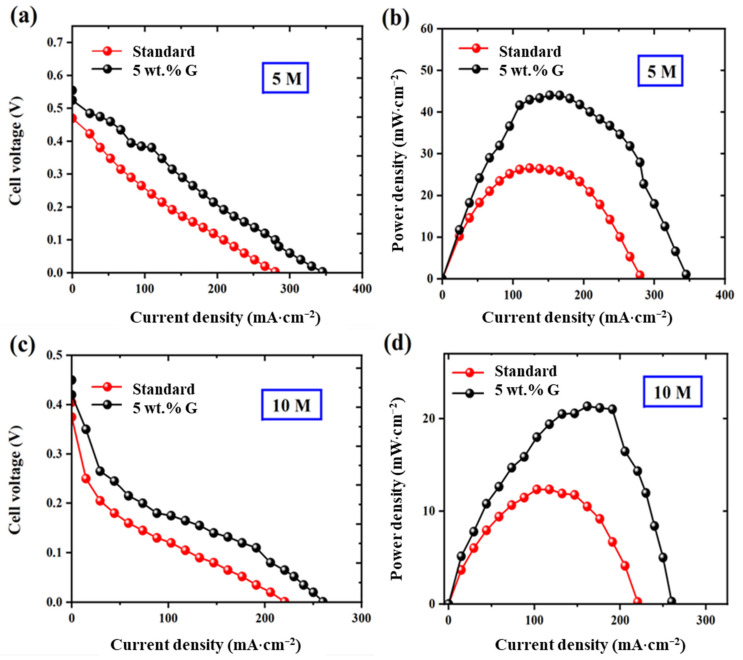
Polarization curve of standard and 5 wt.% graphene MEAs at (**a**,**b**) 5 M concentration and (**c**,**d**) 10 M concentration.

Testing at high methanol concentration is essential, as this can be of great use for comparison to commercial portable sources, henceforth carried out at 5 and 10 M concentrations [7,8,9]. The impact of methanol blockage effect is more pronounced at these high concentrations—showing 56% (45 mW·cm^−2^ peak power density compared to 25 mW·cm^−2^ for the standard) and 75% (22 mW·cm^−2^ peak power density compared to 12 mW·cm^−2^ for the standard) performance improvements at 5 and 10 M concentrations, which are far higher than at 1 M concentration power performance results (36% improvement) as shown in polarization curve (voltage and power density profile) in Figure 8 for standard and 5 wt.% graphene MEAs.

### 2.6. Durability Testing

**Figure 9:**
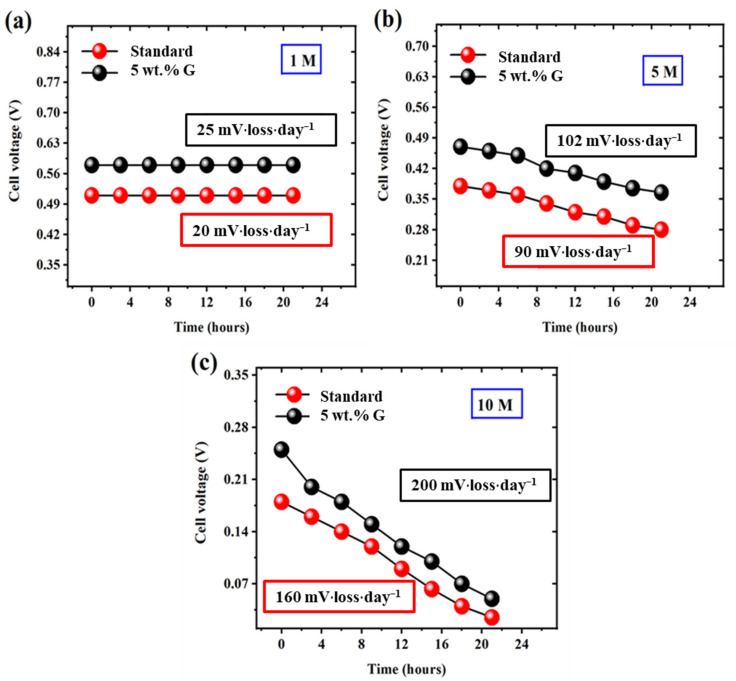
Long term voltage profile of standard and 5 wt.% graphene MEAs at (**a**) 1 M, (**b**) 5 M and (**c**) 10 M methanol concentrations.

**Figure 10:**
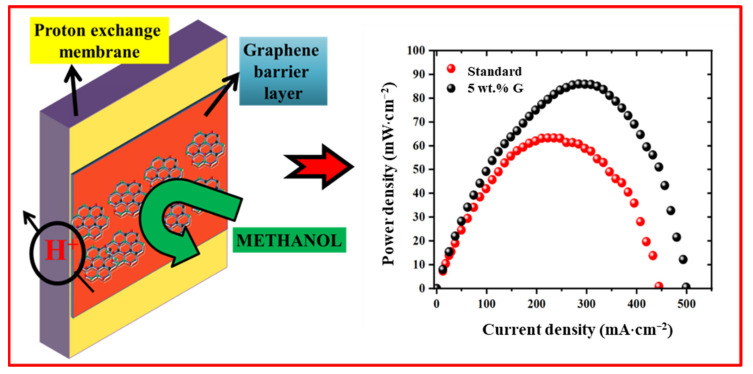
Schematic of impact of addition of graphene nano-layer in membrane region (above the anode catalyst layer) and final fuel cell performance.

Durability testing aids in evaluation of graphene stability for prolonged periods of time henceforth carried out for 20 h at constant current density of 50 mA·cm^−2^. Figure 9 shows the minimal (and similar profile) voltage losses for both 5 wt.% graphene barricade layer and standard MEAs, at different 1 M (Figure 9a), 5 M (Figure 9b) and 10 M (Figure 9c) concentrations confirming their promising nature for long-term power supply applications.

After durability testing, minor reductions in open circuit voltage, peak power density and selectivity values are observed. This is attributed to the dissolution of graphene from the fuel cell electrode due to the high flow rate of incoming fuels. This is evident from top view (Figure S5a,b) and cross-sectional view (Figure S5c,d, Supplementary Materials) of SEM images of electrodes before and after durability testing, showing damaged surface. Since it is beyond the scope of this work, (considering multiple factors—experimental), to analyze the intrinsic behavior of electrodes during fuel cell operation, we have decided to take this for future studies.

### 2.7. Testing in Ethanol and 2-Propanol Fuel Cell Systems

It well-known that graphene prevents ethanol and propanol flow through its channels [13,14,15], hence it is tested at fuel cell systems using these alcohols as fuels. Considerable performance improvements—power density curve (Figures S6a and S7a, Supplementary Materials) are obtained, when tested at 1 M ethanol and 1 M 2-propanol fuel cell systems at wide temperature range (Figures S6b and S7b). These results confirm the versatility of graphene usage in various liquid alcohol-based fuel cells.

Overall, 2D graphene prepared by facile liquid phase exfoliation approach has been utilized as a barrier layer onto the anode side (sprayed above the catalyst layer) due to its imparting high selectivity to the membrane region (fuel passage prevention characteristics) and thereby enhanced improvements in performance in methanol/ethanol/propanol systems are obtained. 

Starting from material characterization results, the high C/O ratio of prepared graphene has aided in enhanced hydrophobicity to the fuel cell electrode, whereas few layer thicknesses resulted in insignificant proton resistance. Combined effect of water/fuel transport on the anode due to changes in electrode crack separation, reduced fuel crossover (methanol permeability, MP), enhanced fuel oxidation on anode side and barrier effect have led to high membrane selectivity. This is depicted in the Figure 10 to summarize the role of graphene on power performance.

Using this at low weight percentages—5 wt.%, has resulted in 40%, 56% and 75% improvement in 1, 5 and 10 M methanol concentrations in addition to enhancements in ethanol and propanol systems. These beneficial effects are also observed at ethanol and propanol fuel cell systems over a wide temperature range (20 °C to 60 °C). Further increasing the graphene weight percentage (stacking), gives poor performance due to the dominant effect of proton transfer resistance affecting selectivity. 

In comparison to the previous work reported (using ozonated graphene, chemical vapor deposited graphene, mechanically exfoliated hexagonal boron nitride materials in the literature) the results obtained in this work offer advantages in various prospects: ease in preparation of graphene, utilization methodology, controllability, short- and long-term performance and diverse usage in various LAFCs, which are given in Table S4, Supplementary Materials. 

Also efforts like improving the efficiency of exfoliation (obtaining lesser thick material than obtained in current conditions, increased 2D material concentration by using solvents closer to the surface energy of graphite—80 mJ·m^−2^, rather than acetone in this work, effect of sonication time, centrifuge speed and duration), studying the effect of flake size, and testing in formic acid, ethanol, hydrogen fuel cells will be of significant interest and be the follow-up of this work.

## 3. Materials and Methods

### 3.1. Graphene Preparation Procedure and Characterization

Graphene from liquid phase exfoliation methodology was prepared according to the procedure by Coleman et al. [36] as follows (given in Figure S8, Supplementary Materials): Graphite flakes (Fischer Scientific, Shanghai, Shanghai municipality, China, size ~325 mesh (40 µm)) at 0.5 mg·mL^−1^ concentration (volume: 20 mL) are dispersed in pure acetone (Fischer Scientific) and bath sonicated (TG16 WS15 L 360 W, 40 kHz bath) for 1 h. The exfoliated dispersion is centrifuged at 5000 rpm for 1 h (Cence TG16-WS model, Xiangyi Centrifuge Instrument Co., Ltd., Changsha, Hunan, China). The supernatant is collected and stored for further testing, whereas thick flakes settled at the bottom are discarded.

Graphene dispersions in acetone were drop-casted (10–20 µL volume) on silicon wafers (MSE Supplies LLC, Tucson, AZ, USA) and dried 48 h in an oven (Quincy lab 10 GC model, Hogentogler and Co. Inc., Columbia, MD, USA) at 200 °C, prior to atomic force microscopy (AFM) (images and surface profiles obtained using CSI nanoprobes in Oxford Instruments, Xuhui District, Shanghai, China), scanning electron microscopy (SEM) (with energy dispersive spectroscopy (EDS) surface morphology analysis/elemental mapping by Hitachi S-3400 equipment, Hitachi High-Tech Corporation, Chaoyang District, Beijing, China) and Raman characterizations (Horiba Scientific with an excitation energy of 532 nm at scanning from 500 to 3000 cm^−1^ Raman shift) studies.

### 3.2. Membrane Electrode Assembly (MEA) Fabrication and Characterization

The MEA was fabricated as described in the procedure [20,41] as follows (Figure S9, Supplementary Materials): First, the gas diffusion media was prepared by spraying carbon ink (microporous layer—MPL) using air-brush (Grainger 48PX91 model, Grainger Inc., Amarillo, TX, USA) onto carbon paper TGPH-090 (280 µm, Fuel Cell Store, College Station, TX, USA) of area 4 cm^2^. The carbon ink contains Vulcan XC-72 (Fuel cell store, College Station, TX, USA) with polytetrafluroethylene (PTFE, 5 wt.% solution, Fuel Cell Store, College Station, Texas, USA) in 10 wt.% in iso-propanol (99% purity, Fischer Scientific). After spray-coating this was placed in an oven (Quincy lab 10 GC model) at 150 °C for 1 h and 70 °C overnight.

Then catalyst ink is sprayed above the MPL, till a platinum loading of 1.5 mg·cm^−2^ for both anode and cathode was reached. Catalyst ink contains catalyst (60 wt.% platinum on Vulcan XC-72 for cathode and 60 wt.% platinum: ruthenium on anode—from Fuel Cell Store) with 15 wt.% Nafion (5 wt.% solution in water, Fuel Cell Store) in acetone (bath sonicated and stirred to obtain a homogeneous ink).

Polymer electrolyte membrane—Nafion 115 (130 µm thickness, Sigma Aldrich, Merck KGaA, Shanghai, Shanghai municipality, China) was pre-treated by heating in de-ionized water (18.2 MΩ cm purity, from Millipore Synergy, Merck KGaA, Pudong New District, Shanghai, China), 10 wt % hydrogen peroxide (98% purity, Fischer Scientific) and 1 M sulphuric acid (98% purity from Fischer Scientific and diluted) solutions at 80 °C for an hour, approximately. Then, this is placed between the anode and cathode electrode and fixed in fuel cell test system at 1.5 N·m pressure using Checkline DTW 100f (Electromatic Equipment Co. Inc., Lynbrook, NY, USA) wrench.

Then, the MEA-containing fuel cell was activated at 60 °C by air-starvation mode (as described in the procedure [42,43]) by passing 10 mL·min^−1^ of 1 M methanol and 250 mL·min^−1^ (2 bar) of dry oxygen (Figure S10, Supplementary Materials, shows the fuel cell set up used in the laboratory), until optimum performance was achieved.

In this work, graphene is sprayed at different weight percentages, 2.5 wt.%, 5 wt.%, 7.5 wt.%, 10 wt.%, 20 wt.% and 25 wt.% (in relation to the weight of Nafion membrane 115 of size 4 cm^2^) on the anode electrode above the catalyst layer. All other materials and deposition methods used in the MEA preparation, remained the same.

Top view morphology, crack size and cross-sectional morphology of fuel cell electrodes—with and without graphene coating were analyzed by Hitachi S-3400 SEM equipment.

### 3.3. Polarization Curve

Polarization curve (cell voltage and power density profile) is obtained by using DC power supply (Nice Power 30 V, 10 A model, Shenzhen Bodunbaili Electronic Co., Ltd., Shenzhen, Guangdong, China) by sweeping current from zero to maximum and recording the voltage by multimeter (Fluke 87 V model, Beijing Fluke Shilu Instrument Maintenance & Service Corp. Ltd., Beijing, China), till the voltage value reaches zero [20,41].

### 3.4. Electrode Porosity (Hydrophobic and Hydrophilic) Measurements

Electrode porosity is obtained by immersing the electrode in pure *n*-decane (Fischer Scientific) solvent, followed by weighing, where porosity is calculated from the difference in weight of electrodes before and after immersion and then divided by the density of n-decane as described in the procedure [37,38].

Hydrophilic porosity is obtained by placing the sample in a lab-made set-up, where the electrode is exposed to incoming water vapor as illustrated in the Figure S11a–c (Supplementary Materials), and water is vaporized by placing the apparatus in an oil bath (Profit Lab Grant 20008 model, Profit Lab GmbH, Landsberger, Berlin, Germany). It is known that following vaporization, the sample weight increases as a result of capillary condensation in the pores. Weight of the sample is measured before and after, to calculate hydrophilic porosity. Hydrophobic porosity is calculated by subtracting hydrophilic porosity from total porosity as described previously in the procedure [38,39].

### 3.5. Fuel Oxidation Propensity (FOP) Measurements

This is obtained by running cyclic voltammetry (using electrochemical work station CHI 660 model, Bioz, Los Altos, CA, USA) by passing fuel (methanol or ethanol or propanol depending on experiments) on the anode (10 mL·min^−1^) and nitrogen (2 bar—200 mL·min^−1^) on the cathode, in the fuel cell (anode side as working electrode and cathode as reference/counter electrode) from 0 to 1 V as described in the procedure [20,42].

### 3.6. Proton Conductivity and Electrode Reaction Resistance (ERR) Measurements

These are obtained by running impedance spectroscopy (potentiostatic mode) in electrochemical work station CHI 660 model, China, in a working fuel cell at 0.4 V, with the cathode side as working electrode and the anode as a reference/counter electrode, in the frequency range from 25 kHz to 0.02 Hz at an amplitude of 5 mV as described in the procedure [20,37]. X-axis intercept of the impedance plot gives the membrane resistance (from where the proton conductivity is calculated) as given in Equation (1) and the arc from x-axis intercept till the completion gives ERR [20,41].

Proton conductivity = T / (R × A)(1)

where, T—Membrane thickness (cm); R—Resistance (ohm) and A—Electrode area. 

### 3.7. Methanol Permeability (MP) Measurements

MP (by electrochemical mode) is obtained by running linear sweep voltammetry and scanning from 0 to 1 V at rate of 5 mV·s^−1^ by passing 10 mL·min^−1^ of methanol on the anode side and passing 200 mL·min^−1^ of nitrogen (2 bar pressure) on the cathode side by connecting the working electrode to the cathode and reference/counter electrode to the anode. The value of current plateau at which methanol oxidation becomes constant is noted as ‘MP current density’ and is carried out as described in the procedure from other work [20,41].

MP (by gravimetric mode) measurement is carried out in the lab-made set up as given in Figure S11a–c (Supplementary Materials), except that methanol is used instead of water, and the weight of the complete set up is plotted as a function of time, to evaluate permeability, as described in the procedure [20]. To evaluate ethanol/2-propanol permeability, methanol is replaced by ethanol and 2-propanol respectively. Different electrode areas are tested to confirm the results.

### 3.8. Electrode Wettability (Contact Angle) Measurements

Electrode wettability (by contact angle) is attained by placing a 20 µL drop of water on a fuel cell electrode and the contact angle was measured between the droplet and the electrode surface after 60 s, using a KRUSS surface analyzer camera [44,45,46].

### 3.9. Water Upatke (WU) Measurements

WU is attained by drying the MEA at 50 °C for 24 h in an oven (for obtaining dry MEA weight—Dry_MEA_) and immersing in water for 24 h (for obtaining wet MEA weight—Wet_MEA_). The difference in weight of the dry and wet sample gives the percentage of water uptake (WU) as given in Equation (2) below. All these measurements are carried out as described in the procedure [47,48].

WU = (Wet_MEA_ − Dry_MEA_) × 100 / (Dry_MEA_) (2)

where, WU—Water uptake percentage (%); Wet_MEA_—Weight of the wet MEA (grams) and Dry_MEA_—Weight of the dry MEA (grams). 

### 3.10. Ion-Exchane Capacity Measurements

Ion-exchange capacity of MEA is attained by immersing MEA in sodium hydroxide solution for 24 h and titration against phenolphthalein. The volume of phenolphthalein utilized until the MEA-immersed solution turns pink and gives ion-exchange capacity of the MEA (Figure S12, Supplementary Materials) [49,50].

## 4. Conclusions

In this work, few-layer graphene flakes prepared by facile, scalable and eco-friendly liquid phase exfoliation (bath sonication) approach of graphite in acetone medium and utilizing them as barrier layer by mounting them next to the membrane on the anode side (sprayed above the catalyst layer) in direct liquid alcohol fuel cells (LAFCs) involving methanol, ethanol and propanol systems is proposed. The material characterizations by atomic force microscopy (AFM), Raman spectroscopy and scanning electron microscopy (SEM), show that graphene is of 2–5 nm thickness and 4 µm uniform lateral size.

The cell performance tests exhibit that 5 wt.% graphene barrier layer, at direct methanol fuel cell (DMFC) testing conditions (60 °C and 1 M methanol), increases the maximum power density from for the standard 63 to 91 mW·cm^−2^. This barrier layer reduces the methanol permeation (MP) from 153 to 112 mA·cm^−2^, with slight decrease in proton conductivity from 101 to 84 mS·cm^−1^, which has led to better methanol utilization—overall improving membrane selectivity (proton conductivity to fuel crossover ratio).

This methanol barrier effect is more visible at highly concentrated methanol operations, where 36% and 75% improvements in fuel cell performance are observed. Long-term testing for 20 h also confirms this effect by displaying minimal voltage losses.

This newly reported work tends to eliminate limitations by using liquid phase exfoliated graphene as a barrier layer due to its feasibility, environmental friendliness and mass production characteristics—yielding few layer nano-materials, posing competition to expensive and complex—chemical vapor deposition (CVD) and mechanical exfoliation methods, in addition to power performance improvements. This also opens new possibilities for other crystals: hexagonal boron nitride (hBN), tungsten di sulphide (WS_2_) and molybdenum di sulphide (MoS_2_) to be used as barrier layers, overall pushing the commercial circle of direct LAFCs.

## Figures and Tables

**Table 1:** Porosity, hydrophobic, hydrophilic porosity and contact angle values of standard and different graphene wt.% electrodes.

Electrode	Porosity (%)	Hydrophobic Porosity (%)	Hydrophilic Porosity (%)	Contact Angle (°)
Standard	73	61	39	121
2.5 wt.%	72	62	38	124
5 wt.%	68	66	34	132
7.5 wt.%	63	67	33	137
10 wt.%	58	69	31	144
20 wt.%	55	71	29	149
25 wt.%	54	73	27	162

**Table 2:** Methanol permeability, proton conductivity and membrane selectivity values of standard and different graphene wt.% MEAs.

MEA	Fuel—Methanol Permeability (mp, Electrochemical Mode) (ma·cm^2^)	Proton Conductivity (ms·cm^−1^)	Membrane Selectivity (ms·cm·ma^−1^)
Standard	155	103	0.65
2.5 wt.%	139	91	0.66
5 wt.%	112	87	0.77
7.5 wt.%	101	72	0.71
10 wt.%	93	63	0.67
20 wt.%	88	56	0.63

## Data Availability

The datasets used and/or analyzed in the present study are available from the corresponding author upon reasonable request.

## Supplementary Materials

The following supporting information can be downloaded at: https://www.mdpi.com/article/10.3390/molecules27093044/s1, Figure S1: Raman spectrum of graphite and graphene, dropcasted on silicon wafer; Figure S2: (**a**) Cell voltage; (**b**) Power density profile obtained at 60 °C in 1 M methanol/oxygen conditions for standard and 2.5 wt.% graphene MEAs; Figure S3: (**a**) Methanol permeability (by gravimetric mode) at 1 M methanol condition and (**b**) Wettability (contact angle) for standard and graphene 5 wt.% electrodes; Figure S4: Peak power density values for standard and 5 wt.% graphene MEAs at different temperatures; Figure S5: SEM images (**a**,**b**—top-view and **c**,**d**—cross-sectional) of electrodes coated with 5 wt.% graphene before and after durability testing; Figure S6: (**a**) Power density curve and (**b**) temperature profile of standard and 5 wt.% graphene MEAs at 1 M ethanol concentration; Figure S7: (**a**) Power density curve and (**b**) Temperature profile of standard and 5 wt.% graphene MEAs at 1 M 2-propanol concentration; Figure S8: Graphene dispersion preparation procedure; Figure S9: Membrane electrode assembly (MEA) preparation methodology; Figure S10: Single fuel cell set up used in the laboratory; Figure S11: (**a**). Schematic of electrode porosity measurement; (**b**) Lab-made set up; (**c**) Closer view of the electrode arrangement; Figure S12: Determination of ion-exchange capacity experiment - (**a**) Before and (**b**) After phenolphthalein addition; Table S1. Average values of elemental analysis results of graphene flakes by elemental dispersive spectroscopy (EDS); Table S2. Comparison of water-uptake (WU) and ion-exchange capacity values of different electrodes; Table S3. Open circuit voltage, and peak power density values of standard and different graphene wt.% MEAs; Table S4. Comparison of this work with recent literature. References [51,52,53] are cited in the Supplementary Materials.
