# Supplementary material for: Nano-Graphene Layer from Facile, Scalable and Eco-Friendly Liquid Phase Exfoliation Strategy as Effective Barrier Layer for High-Performance and Durable Direct Liquid Alcohol Fuel Cells
Source: Molecules. 2022 May 9;27(9):3044. doi: 10.3390/molecules27093044 (PMC9100842; doi:10.3390/molecules27093044)
Supplement: Supplementary file 1 [file molecules-27-03044-s001.zip › molecules-1676546-supplementary.pdf]

molecules

MDPI

Supplementary Materials

Nano-Graphene Layer from Facile, Scalable and Eco-Friendly Liquid Phase Exfoliation Strategy as Effective Barrier Layer for High-Performance and Durable Direct Liquid Alcohol Fuel Cells

Prabhuraj Balakrishnan [1], Fereshteh Dehghani Sanij [1], Zhixin Chang [1], P.K. Leung [2], Huaneng Su [1], Lei Xing [3] and Qian Xu [1,*]

[1] Institute for Energy Research, Jiangsu University, Zhenjiang 212013, China; prabhuraj@ujs.edu.cn (P.B.); dehghan82@gmail.com (F.D.S.); zhixinchang1996@163.com (Z.C.); suhuaneng@ujs.edu.cn (H.S.)

[2] MOE Key Laboratory of Low-grade Energy Utilization Technologies and Systems, Chongqing University, Chongqing 400030, China; leungpuiki@hotmail.com

[3] Department of Chemical Engineering, Loughborough University, Loughborough LE11 3TU, UK; xinglei1314@gmail.com

* Correspondence: xuqian@ujs.edu.cn

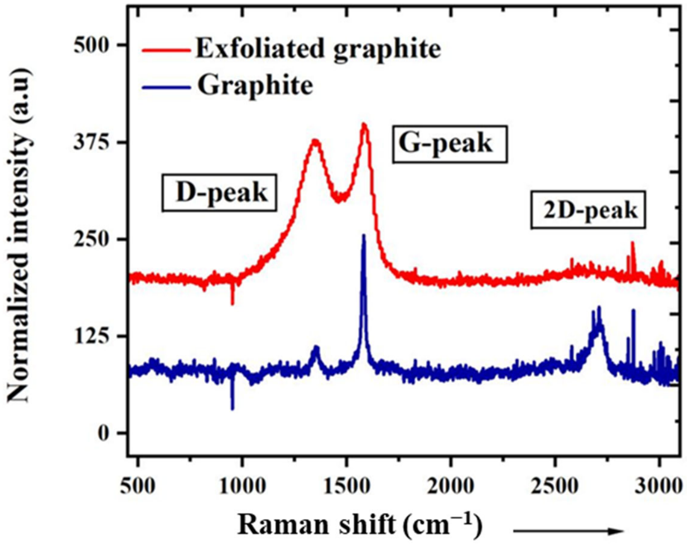

Figure S1. Raman spectrum of graphite and graphene, dropcasted on silicon wafer.

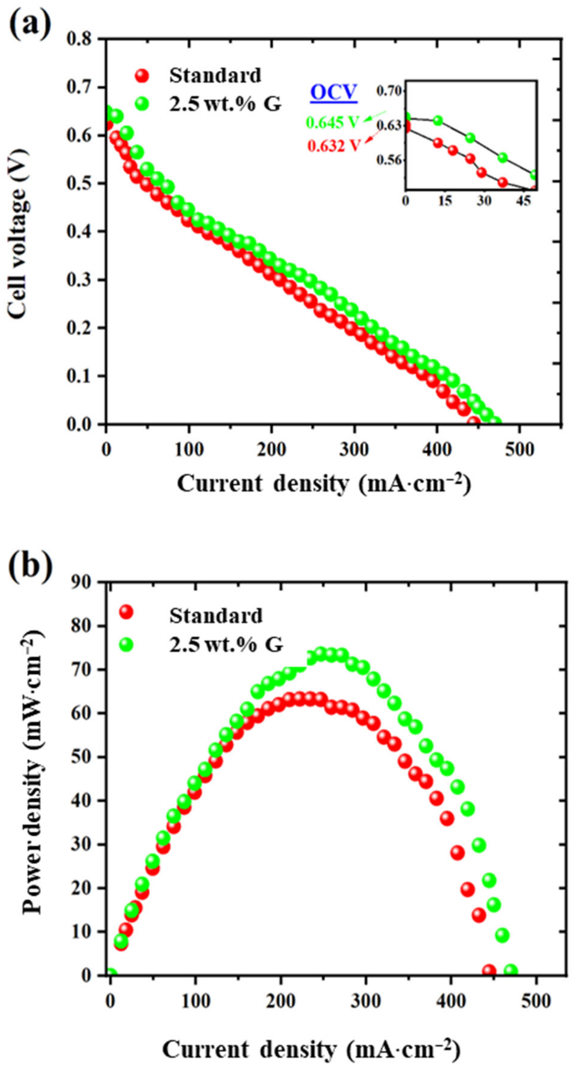

Figure S2. (**a**) Cell voltage; (**b**) Power density profile obtained at 60 °C in 1 M methanol/oxygen conditions for standard and 2.5 wt.% graphene MEAs.

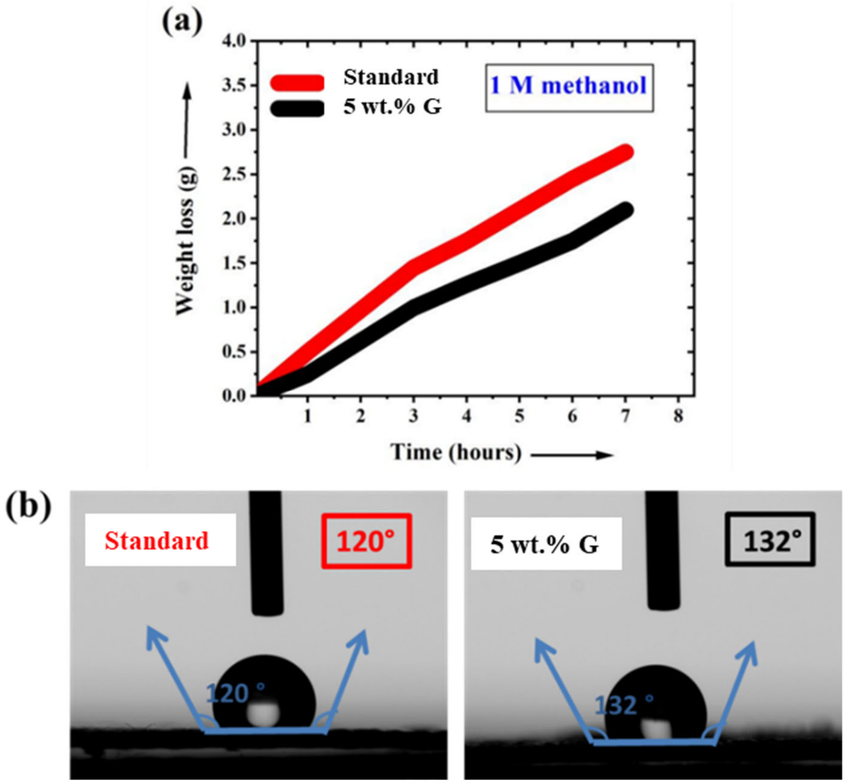

Figure S3. (**a**) Methanol permeability (by gravimetric mode) at 1 M methanol condition and (**b**) Wettability (contact angle) for standard and graphene 5 wt.% electrodes.

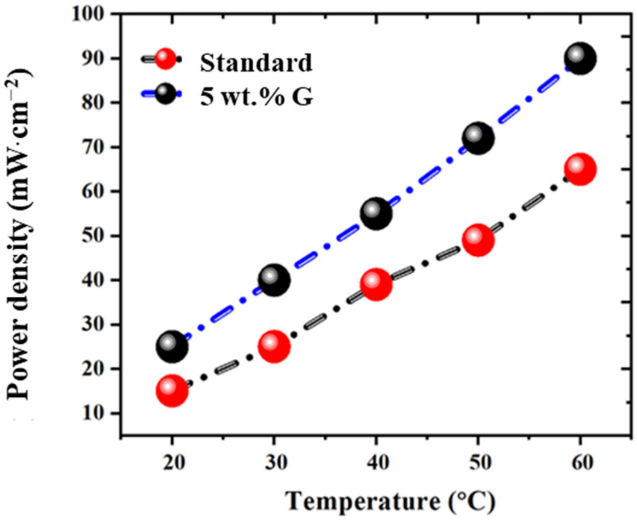

Figure S4. Peak power density values for standard and 5 wt.% graphene MEAs at different temperatures.

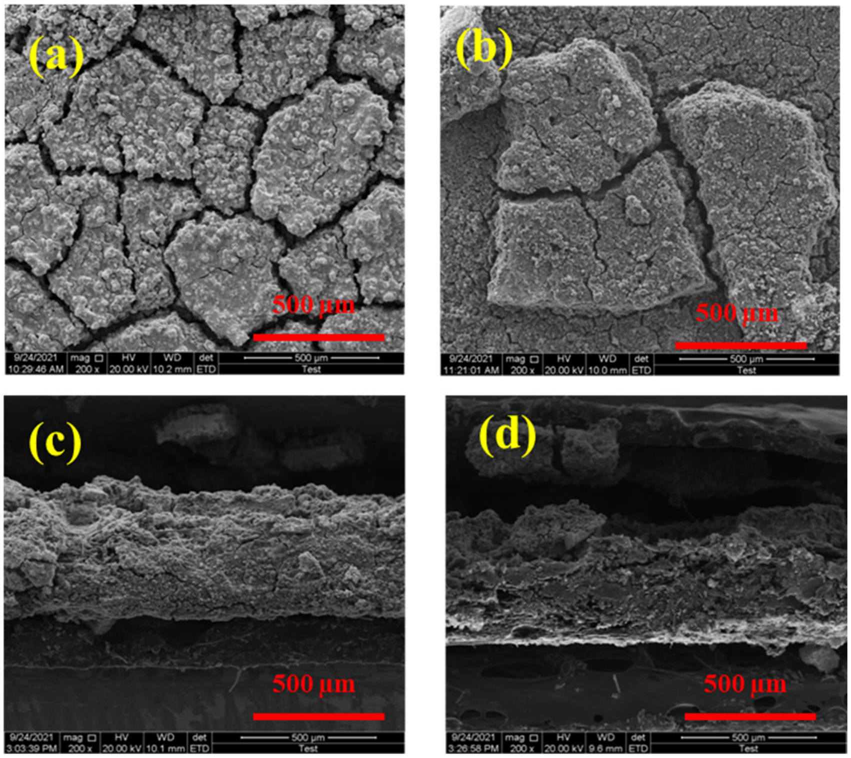

Figure S5. SEM images (**a**, **b** - top-view and **c**, **d** - cross-sectional) of electrodes coated with 5 wt.% graphene before and after durability testing.

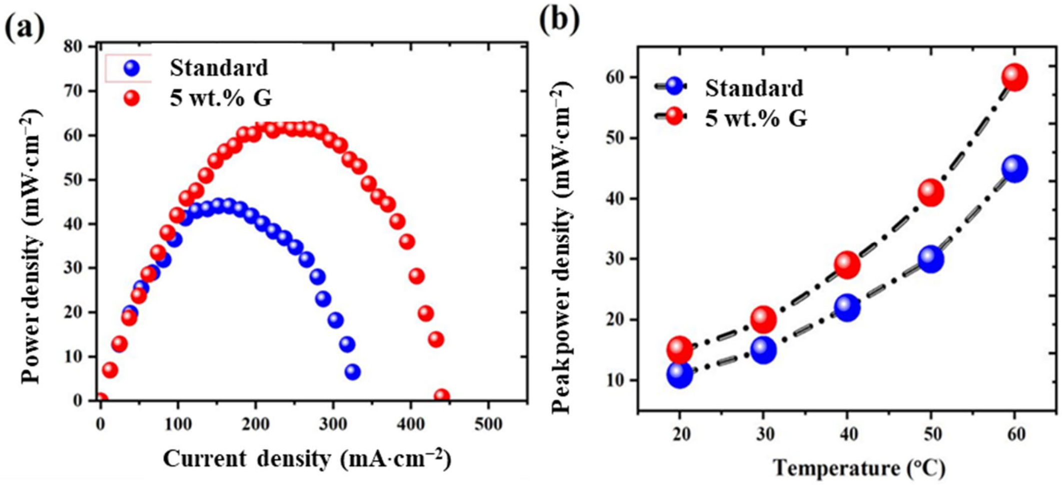

Figure S6. (**a**) Power density curve and (**b**) temperature profile of standard and 5 wt.% graphene MEAs at 1 M ethanol concentration.

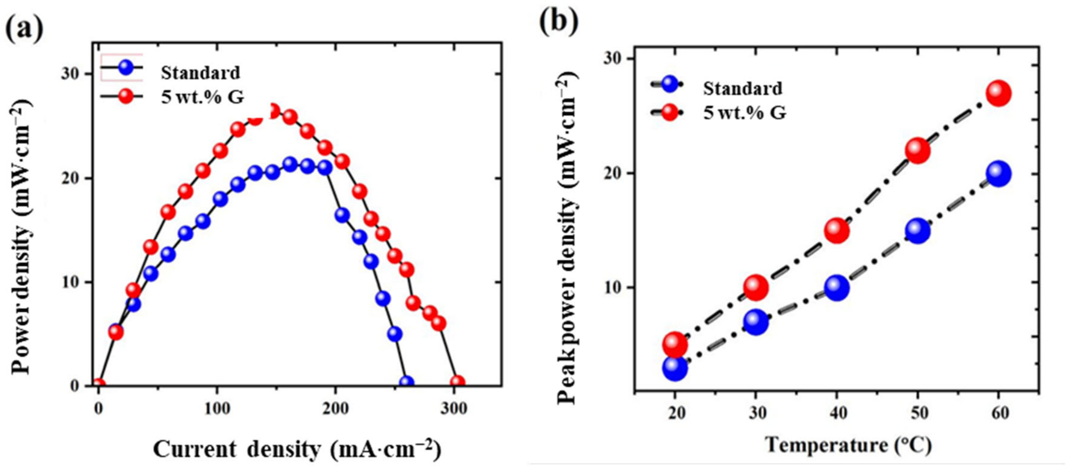

Figure S7. (**a**) Power density curve and (**b**) Temperature profile of standard and 5 wt.% graphene MEAs at 1 M 2-propanol concentration.

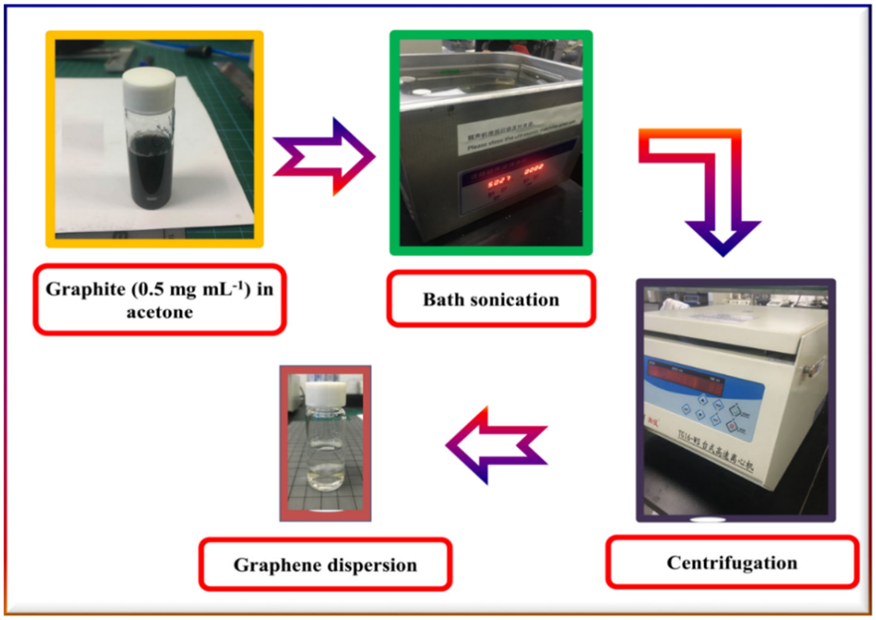

Figure S8. Graphene dispersion preparation procedure.

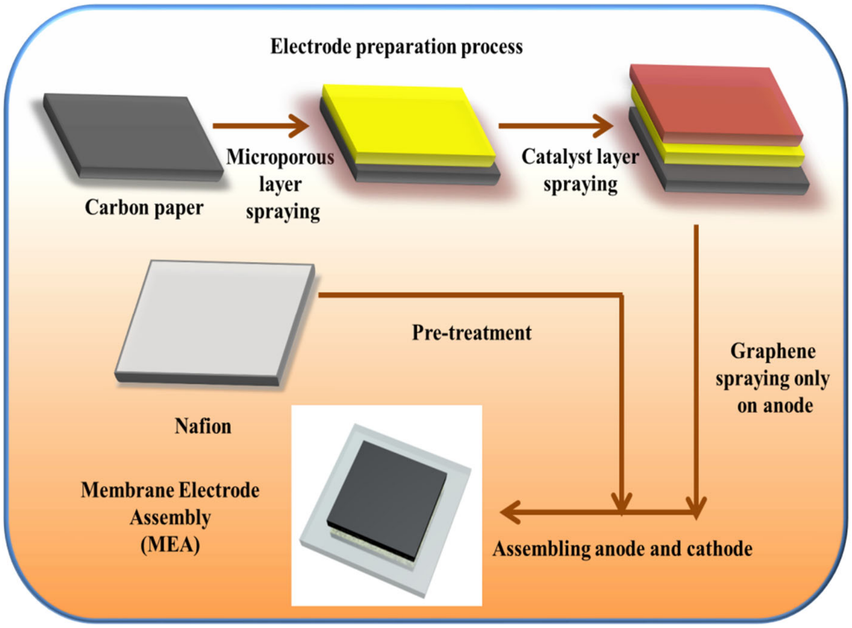

Figure S9. Membrane electrode assembly (MEA) preparation methodology.

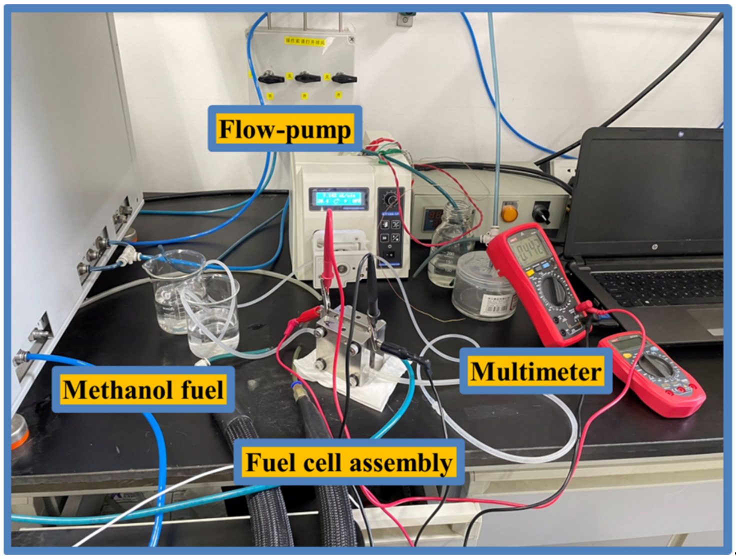

Figure S10. Single fuel cell set up used in the laboratory.

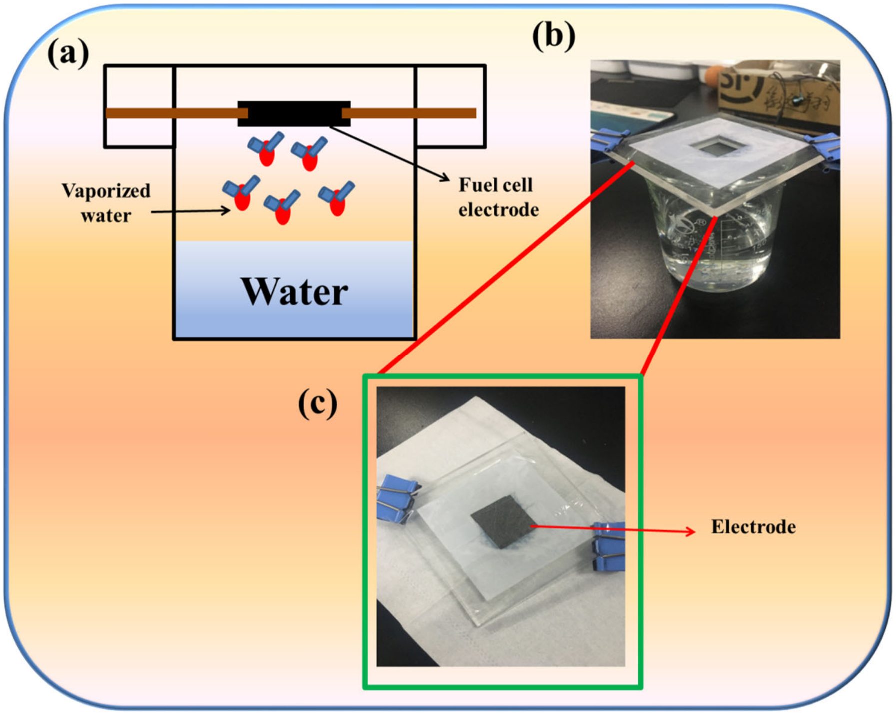

Figure S11. (**a**). Schematic of electrode porosity measurement; (**b**) Lab-made set up; (**c**) Closer view of the electrode arrangement.

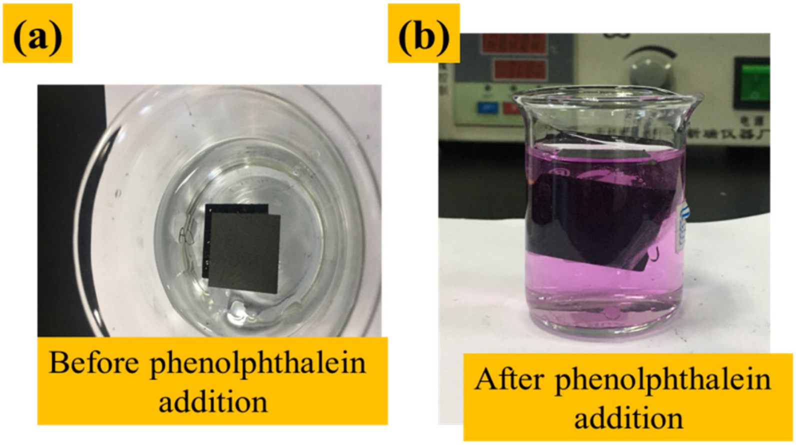

Figure S12. Determination of ion-exchange capacity experiment - (**a**) Before and (**b**) After phenolphthalein addition.

Table S1. Average values of elemental analysis results of graphene flakes by elemental dispersive spectroscopy (EDS).

GRAPHENE FLAKE	WEIGHT PERCENTAGE (wt.%)
**Carbon**	**82**
**Oxygen**	**18**

Table S2. Comparison of water-uptake (WU) and ion-exchange capacity values of different electrodes.

ELECTRODE	WATER UPTAKE (WU) (%)	ION-EXCHANGE CAPACITY (%)
Standard	--	--
2.5 wt.%	−6	4
5 wt.%	−7	5
7.5 wt.%	−10	11
10 wt.%	−22	18
20 wt.%	−24	21
25 wt.%	−32	27

Table S3. Open circuit voltage, and peak power density values of standard and different graphene wt.% MEAs.

MEA	OPEN CIRCUIT VOLTAGE (V)	PEAK POWER DENSITY (mW cm^{-2})
Standard	0.632	63
2.5 wt.%	0.645	73
5 wt.%	0.660	91
7.5 wt.%	0.595	61
10 wt.%	0.550	45
20 wt.%	0.500	29

Table S4. Comparison of this work with recent literature.

MATERIAL	PREPARATION METHODOLOGY	PERFORMANCE RESULTS AND CONDITIONS	REFERENCE
**Graphene-1,4 phenyl diamine hydrochloride (PDHC)**	50 layered arrangement of graphene oxide - PDHC.	Maximum performance of 65 mW cm^{-2} compared to showing 35 mW cm^{-2} reporting by reducing crossover.	Wang et al. 2015 [51]
**Ozonated graphene (OG)**	Graphene is exposed to ozone gas	Nafion-OG-Nafion membrane shows 180 mW·cm^{-2} compared to Nafion-G-Nafion performance of 120 mW·cm^{-2}	Gao et al. 2014 [52]
**Hexagonal nitride (hBN)**	hBN by mechanical exfoliation	Spincoated hBN on Nafion showed reduced fuel (hydrogen) permeability reported stable open circuit voltage (OCV)	Lee et al. 2019 [53]
**Graphene**	Chemical vapour deposited (CVD) graphene is transferred onto fuel cell electrode	45% improvement in performance are reported	Holmes et al. 2016 [20]
**Graphene**	CVD graphene sandwiched between two Nafion 212	120% performance improvement at high concentration methanol passive fuel cell systems	Yan et al. 2016 [21]
**Graphene**	Graphene dispersion from XFNano Inc is sprayed onto anode	82% improvement in performance are reported at 8 M methanol fuel cell conditions	Xu et al. 2020 [22]
**Graphene**	Graphene prepared by liquid phase exfoliation and added as barrier layer	36% improvement in methanol fuel cell performance are reported inaddition to ethanol and propanol systems	This work

References for Supporting Information

20. Holmes, S.M.; Balakrishnan, P.; Kalangi, V.S.; Zhang, X.; Lozada-Hidalgo, M.; Ajayan, P.M.; Nair, R.R. 2D Crystals Significantly Enhance the Performance of a Working Fuel Cell. *Adv Energy Mater* **2016**, *7*, 1601216.
21. Yan, X.; Wu, R.; Xu, J.; Luo, Z.; Zhao, T. A monolayer graphene–Nafion sandwich membrane for direct methanol fuel cells. *J. Power Sources* **2016**, *311*, 188–194.
22. Xu, Q.; Sun, W.; Zhang, J.; Zhang, W.; Ma, Q.; Su, H.; Xing, L. Constructing a graphene-contained layer in anode to improve the performance of direct methanol fuel cells using high-concentration fuel. *Int. J. Green Energy* **2020**, *18*, 566–577.
51. Wang, L.; Lai, A.; Lin, C.; Zhang, Q.; Zhu, A.; Liu, Q. Orderly sandwich-shaped graphene oxide/Nafion composite membranes for direct methanol fuel cells, *J. Membr. Sci.* **2015**, *492*, 58–66.
52. Gao, W.; Wu, G.; Janicke, M.; Cullen, D.; Mukundan, R.; Baldwin, J.; Brosha, E.; Galande, C.; Ajayan, P.; More, K.; et al. Ozonated Graphene Oxide Film as a Proton-Exchange Membrane, *Angew. Chem., Int. Ed.* **2014**, *53*, 3588–3667.
53. Lee, S.; Jang, W.; Kim, M.; Shin, J.; Park, H.; Jung, N.; Whang, D. Rational Design of Ultrathin Gas Barrier Layer via Reconstruction of Hexagonal Boron Nitride Nanoflakes to Enhance the Chemical Stability of Proton Exchange Mem-brane Fuel Cells, *Small Methods*. **2019**, *15*, 1903075.
